# Seed Germination Indicates Adaptive Transgenerational Plasticity in a Submerged Macrophyte

**DOI:** 10.3389/fpls.2018.01592

**Published:** 2018-11-21

**Authors:** Hong Su, Tianshun Zhu, Xiaohu Bai, Leyi Ni, Ping Xie, Xiaolin Zhang

**Affiliations:** ^1^Donghu Experimental Station of Lake Ecosystems, State Key Laboratory of Freshwater Ecology and Biotechnology, Institute of Hydrobiology, Chinese Academy of Sciencess, Wuhan, China; ^2^University of Chinese Academy of Sciences, Beijing, China; ^3^College of Life Sciences, Zaozhuang University, Zaozhuang, China; ^4^Xinjiang Academy of Environmental Protection Science, Ürümqi, China

**Keywords:** transgenerational plasticity, submerged macrophyte, seed germination, water depth, adaptive characters, trade-offs

## Abstract

Adaptive transgenerational plasticity is an important evolutionary strategy in plants. We investigated the resource allocation strategy in sexual reproduction and performed an *in situ* seed germination experiment of *Potamogeton maackianus* to reveal their responses to different water depths. Later, we discussed the biased adaptability to the maternal habitat in this species. We found a positive correlation between sexual and asexual reproduction in water depths from 1.0 m to 3.0 m, such a correlation failed to occur in 4.0 m water depth. These results indicate that the trade-off between sexual and asexual reproduction should only be expected in a stressful habitat, where resource acquisition is limited. For trade-off between quantity and quality of sexual units in different water depths, *P. maackianus* tends to produce more but lower quality sexual reproductive units in shallow water, and fewer but higher quality sexual units are found in deep water. The total germination percentage of seeds of *P. maackianus* was relatively poor, less than 46.65% in all of the treatments. The maximum germination percentage of seeds from 1.0 m, 2.0 m, 3.0 m, and 4.0 m water depths are 14.4%, 17.75%, 25.51%, and 46.65%, respectively. Seeds with higher germination percentage were from deeper water depths. The most interesting result was that the maximum final germination percentage occurred only when treatment water depth was the same as collection water depth. Our result showed that the variations in germination characters of the studied species appear to be based partly on the effects of maternal environmental factors. Our findings proved the adaptive transgenerational plasticity in *P. maackianus*, which will play an important role in evolutionary response to the selection of water depths.

## Introduction

Acquisition and maintenance of adaptability is essential for the evolution of plants. Some of the adaptive variations in response to particular environmental stresses could be inherited by the offspring from their maternal individuals, and these variations could enhance offspring fitness under the same environmental stress (Herman and Sultan, 2011). Such adaptive transgenerational plasticity due to maternal environments is common in terrestrial plants (Galloway, 1995, 2001a,b, 2002, 2005; Galloway and Etterson, 2007). Recently, adaptive transgenerational plasticity has been re-considered as a potential source of ecologically and evolutionarily meaningful variations (Roach and Wulff, 1987; Sultan, 1996; Donohue and Schmitt, 1998). The importance of adaptive transgenerational plasticity was considered to be related to population maintenance, evolutionary process, and species invasion (Herman and Sultan, 2011). This heritable plastic response to the environment may play a central role in the process of evolution in plants.

However, most of the case studies and reviews of adaptive transgenerational plasticity are from terrestrial plants (Galloway, 1995, 2001a,b, 2002, 2005; Latzel and Klimešová, 2010; Herman and Sultan, 2011), although aquatic plants (especially submerged macrophytes) play a very important role in freshwater ecosystems (Carpenter and Lodge, 1986; Scheffer, 1990; Jeppesen et al., 1998). Until now, adaptive transgenerational plasticity and maternal effects have been rarely reported in true aquatic plants. Several studies indicated local adaptation in some aquatic plants (Purohit and Singh, 1987; Hangelbroek et al., 2003; Iida et al., 2007; Richards et al., 2011; Xie et al., 2015), but their experimental design and results failed to directly prove adaptive transgenerational plasticity. Among the former studies, Li et al. (2015) indicated the potential relationship between seed behavior and maternal environments of *Potamogeton pectinatus*, but their study lacked *in situ* experimental support.

The rare report of adaptive transgenerational plasticity in aquatic plants could be attributed to two main reasons. The first reason is the longtime overlooking of sexual reproduction in aquatic plants. Although asexual reproduction (vegetative reproduction) is often assumed to be the dominant mode of reproduction in aquatic plants (Sculthorpe, 1967; Hutchinson, 1975), sexual reproduction continues to play a central role in the population biology of these plants (Philbrick and Les, 1996). Based on recent studies (Hangelbroek et al., 2002, 2003; Li et al., 2004; Chen et al., 2006; Triest and Fenart, 2014), the frequency of sexual reproduction in aquatic plants has been overlooked for a long time. The second reason is the common conclusion that the water habitat is more stable than the terrestrial habitat, which is based on the following facts: water exhibits greater chemical and thermal stabilities than air and buffers against many types of catastrophic disturbances; and the convergent evolution in aquatic plants (Sculthorpe, 1967; Philbrick and Les, 1996). However, the water habitat is never more stable than the terrestrial one but the unstability of water habitat displays in some other aspects. Unlike the terrestrial habitat, temperature and moisture are relatively constant in a water body, but other factors change rapidly along with water depth, such as underwater light intensity, current velocity, and dissolved oxygen. There are many heterogeneous environmental niches within the same water body and such heterogeneities are predictable for aquatic plants. Consequently, adaptive transgenerational plasticity should also be expected in aquatic plants.

Among the many environmental factors in water habitats, water depth is the most important one because it affects underwater light intensity and O_2_ availability, shapes characters of individual plants and population, affects assembly of community, and also affects reproductive allocation (Spence, 1982; Maberly, 1993; Wantzen et al., 2008; Fu et al., 2012; Li et al., 2017). Submerged macrophytes are directly under the selective pressure of water depth. The adaptation to water depth is vital to their survival. However, most submerged macrophytes are not restricted to a fixed water depth but usually spread widely along water depth gradient and assemble various structured communities. The understanding of how they adapt to different water depths and how these adaptive characters can be inherited by their offspring is important in evolutionary research of aquatic plants.

*Potamogeton maackianus* is a typical submerged macrophyte widely distributed in East and Southeast Asia (Wiegleb and Kaplan, 1998; Flora of China, Vol. 23) especially in the watershed of the Yangtze River, before eutrophication. It is considered as an indicator species for water quality (Ni, 2001; Fu et al., 2013; He et al., 2015). In many freshwater shallow lakes, *P. maackianus* colonizes large areas and forms dense populations. This is because of its relatively wide ecological niche and high plasticity. According to our former studies, *P. maackianus* has dramatic plastic variation in morphology and biomass allocation (such as stem length, leaf length, branching pattern, specific leaf area, root-shoot biomass ratio, and growth rate) in response to changes in water depth (Fu et al., 2013, 2014). Because there are no specialized turions in this species (Wiegleb and Kaplan, 1998), rhizomes and seeds are the main means of population maintenance and expansion. The main pollination type of this species is anemophily (Jin and Guo, 2001; Zhang et al., 2009). With bisexual flowers, outcrossing and selfing can both be expected. Seeds represent the link between maternal parent and offspring, and seed germination is the first stage of the plant life cycle where natural selection can operate. Therefore, we chose *P. maackianus* as the material and seed germination speed and proportion as principal variables to study potential adaptive transgenerational plasticity.

To find the relationship between water depth (selective pressure) and seed germination (responses) and further discuss the potential transgenerational plasticity, we investigated reproductive allocation and designed an *in situ* germination experiment in response to different water depths to test three hypotheses: (1) Water depth will affect reproductive allocation by means of seed quality, and such affects could be represented by seed germination; (2) Since water depth is an environmental pressure in the germination of seeds, the lowest germination percentage could be found in the deepest water habitat; and (3) The responses of seed germination to different water depths could be related to the habitat in which the seeds mature.

## Materials and Methods

### Studied Species and Experimental Site

*Potamogeton maackianus* is always submerged, and its flowering time is from May to August while blooming time is in July in South China. Three small bisexual flower whorls arranged oppositely on the spica, at the tip of each branch, are found in *P. maackianus*. In each bisexual flower, there are two carpels. Although the pollination type of *P. maackianus* is anemophilous, the stigma can be self-pollinated by pollen grains moving through the air bubble around the inflorescence if the flower is underwater when blooming (Jin and Guo, 2001; Zhang et al., 2009).

**FIGURE 1:**
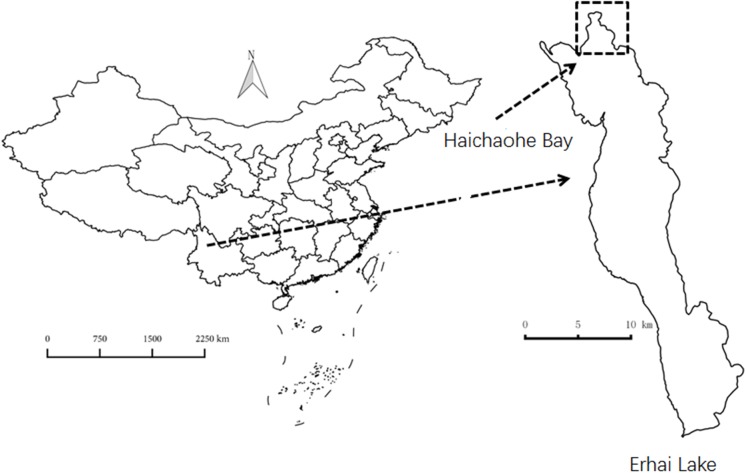
The map shows the location of Erhai Lake and experimental site.

Erhai Lake (25°52′N, 100°06′E) is a freshwater lake located in Yunnan Province, Southwest China. The lake is characterized by a surface area of 250 km^2^ (when water level is 1974 m above sea level), with a maximum water depth of 21 m and an average depth of 11 m. Most of the macrophytes inhabit and dominate the shallow water area (0–3.0 m depth), and only a few species such as *P. maackianus* and *Vallisneria natans* colonize deeper water areas. The population of *P. maackianus* presents a zonation from shoreline to about a 5-m depth in this lake. Haichaohe bay is the biggest bay in the northern part of the lake with an area of 10 km^2^. In this bay, the population of *P. maackianus* has maximum density and is at its deepest distributed limitation (about 5 m). Haichaohe bay was thus used for material collection and for *in situ* germination experiments. The location of Erhai Lake and Haichaohe bay are shown in Figure 1.

### Material Collection

Three kinds of materials were collected from July to September 2016: mature inflorescences with undehisced anthers for pollen counting; ripe seeds for germination experiments; and individuals with ripe seeds for the investigation of resource allocation in sexual reproduction. In this study, we defined a complete shoot with only one internode of stolon as an individual. All the three kinds of materials were collected along the gradient of water depth in Haichaohe bay. Four water depths (1.0 m, 2.0 m, 3.0 m, and 4.0 m) were defined as collection water depths (CWDs) CWD1, CWD2, CWD3, and CWD4, respectively.

In each CWD, 15 individuals with mature inflorescence were randomly collected in July for pollen counting. At least 15 whole individuals with ripe seeds were randomly collected from each CWD in August and September for the study of sexual reproduction allocation traits. The distance between adjacent individuals was maintained as at least 10 m to avoid collecting from the same genet. Ripe seeds, as many as possible, were randomly collected from all CWDs for the germination experiments.

### Sexual Reproduction Traits and Current Velocity Measurement

The following traits were recorded: pollen amount per inflorescence; inflorescence number; seed amount per individual; single seed weight; seed biomass per individual; seed set; and shoot biomass. Because *P. maackianus* never forms specialized asexual organisms such as tubers and turions, we treated the biomass of vegetative parts (shoot biomass) as asexual reproductive allocation. We also calculated seeds/shoot biomass ratio as sexual reproductive resource allocation proportion. In total, 60 inflorescence (15 inflorescence for each CWD) for pollen counting were fixed in FAA fixative solution [constituted of formalin (37–40%), acetic acid, and alcohol (50%) at a ratio of 5: 5: 90 by volume] after collection. All the 12 anthers (there are four anthers in every single flower and three flowers in one inflorescence) were removed from each inflorescence under a dissecting microscope and prepared to count pollen grains. The methods of manual counting of pollen grains followed the method described by Kearns and Inouye (1993). A total 60 individuals (from four CWDs) for the investigation of reproduction allocation traits were washed using tap water to remove the attached algae. All the individuals were separated into vegetative parts (shoots) and seeds. Only ripe seeds were used for the investigation. The number of seeds of each individual was counted. After that, seeds and shoots were dried in an oven at 80°C for 48 h to constant weight. Later, seed biomass and shoot biomass were measured using an analytical balance.

Because seed dispersal distance is closely related to lake current velocity, we used River Surveyor M9 (SonTek) to measure the current velocity of an entire section in Haichaohe bay in October (seed dispersal period).

### *In situ* Germination Experiment

About 2000 ripe seeds were collected randomly from the four CWDs for the germination experiment. After collection, the seeds were washed roughly using tap water to remove algae and then stored at 4°C in darkness until the beginning of the experiments in March 2017 to break the dormancy of the seeds (Hay et al., 2008).

**FIGURE 2:**
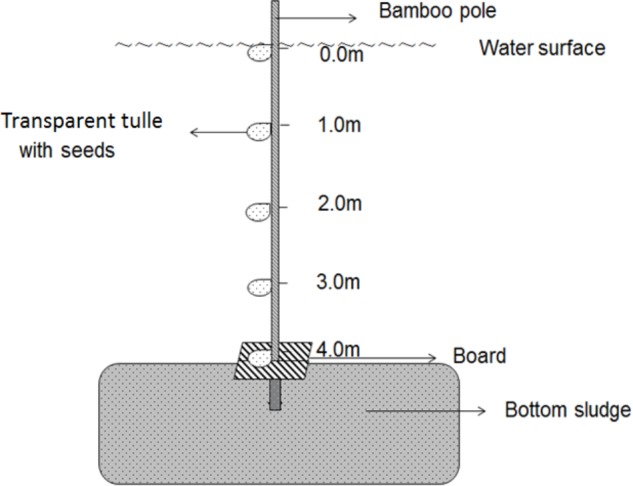
Design of the germination experiment device.

The *in situ* germination experiment was conducted in the area where water depth is 4 m in Haichaohe bay. The treatment water depth (TWD) for the *in situ* experiment was defined as five water depths (0.0 m, 1.0 m, 2.0 m, 3.0 m, and 4.0 m). Three bamboo poles (6 m in length) were used as supports for the *in situ* experiment. Firstly, we made five marks according to the five TWDs on the pole, and the intervals were kept as 1-m. Later, according to the four CWDs, four bags (7 cm × 9 cm) made of transparent tulle were tied at each mark, and each bag contained 30 seeds from each CWD. The CWD of seeds was signed outside the bags. A square board was fixed on the pole just below the lowest mark (TWD4). Subsequently, we inserted the pole into the sediment until the square board was just on the mud surface and made sure the top mark (TWD0) was just on the water surface. The design of the germination device is shown in Figure 2. Three replications were made in this germination experiment. We checked the bags every 3 days to count the newly germinated seeds. After counting, germinated seeds were removed from the bags. A seed was considered as germinated if the radicle that extended from the seed coat was as long as the seed diameter. The experiment was continued until no more seeds germinated over 10 consecutive days.

The *in situ* experiment started on March 15, 2017 and lasted 45 days. During the experiment, Secchi depth (SD), water temperature (T), pH, dissolved oxygen concentration (DO), and underwater photosynthetic active radiation (PAR) were measured near the poles. The SD was measured by a 30-cm diameter Secchi-disk. The parameters of T, DO, and pH were measured using a multifunctional YSI meter (Yellow Springs Instruments, OH, United States). The PAR was measured using an underwater radiation sensor (UWQ-8342) connected to a data logger (Li-1400; LI-COR Company, Lincoln, NE, United States). The extinction coefficient of water was calculated using PAR in different water depths. All the environmental parameters were measured every 5 days over the experimental period.

### Data Analysis

One-way analysis of variance (ANOVA) was used to evaluate the variation of sexual reproduction traits among different CWDs. Pearson’s correlation analysis was performed among the eight reproduction characters to test the covariation of trade-offs. Two-way ANOVA was used to examine the effects of CWD and TWD on seed germination in the *in situ* germination experiment. The final germination percentage was square root transform and treated as dependent variable, while TWD and CWD were treated as independent variables. Post hoc comparisons for all analyses were made with Tukey’s HSD test. Significant differences were determined when *p* < 0.05 or *p* < 0.01.

In many related studies, the time required for half final germination (*T*_50_) was considered as a good estimator to describe the germination speed because *T*_50_ is less affected by a small number of seeds having very long germination times (Thornley, 1986; Li et al., 2000; Jian et al., 2003). It can be derived from a logistic equation:

(1) $$G=\frac{k}{1+a\,\exp\left(-rT\right)}$$

where *G* is the percentage of germination at time *T*; *k* is the maximum germination capacity (observed final germination percentage); and *a* and *r* are estimated parameters. From the fitted equations, the time required for half final germination (*T*_50_) in each treatment can be calculated by the following equation:

(2) $$T_{50}=\frac{In\,a}{r}$$

After calculating *T*_50_, a linear regression analysis was performed between 1/*T*_50_ and water depth to get the potential maximum germination depth (PMGD) in this lake.

All statistical analyses were carried out with SPSS version 22.0.

## Results

### Sexual and Asexual Reproduction Allocation

**FIGURE 3:**
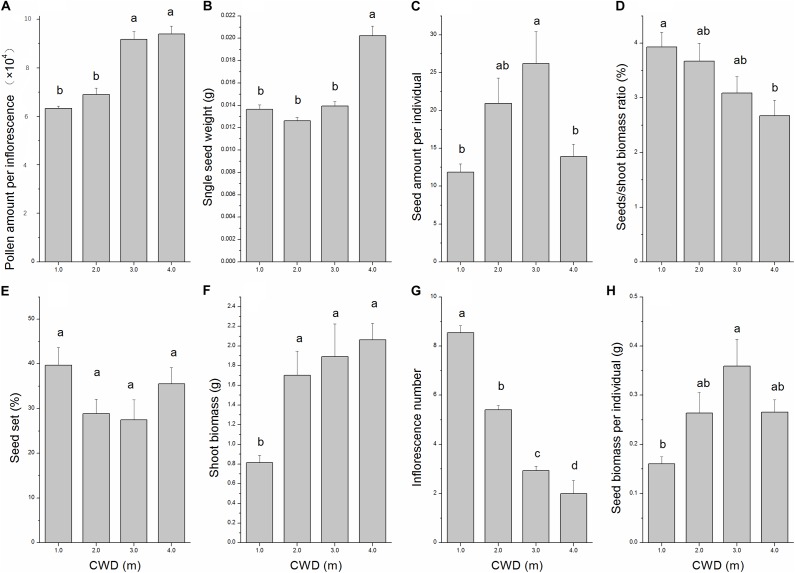
Variation in eight characters depending on water depth analyzed by one-way ANOVA. **(A)** Pollen amount per inflorescence, **(B)** Single seed weight, **(C)** Seed amount per individual, **(D)** Seeds/shoot biomass ratio, **(E)** Seed set, **(F)** Shoot biomass, **(G)** Inflorescence number, and **(H)** Seed biomass per individual. Error bars mean standard error. Turkey’s HSD test was used for *post hoc* comparisons. Different letters indicate significant differences (*p* < 0.05).

**FIGURE 4:**
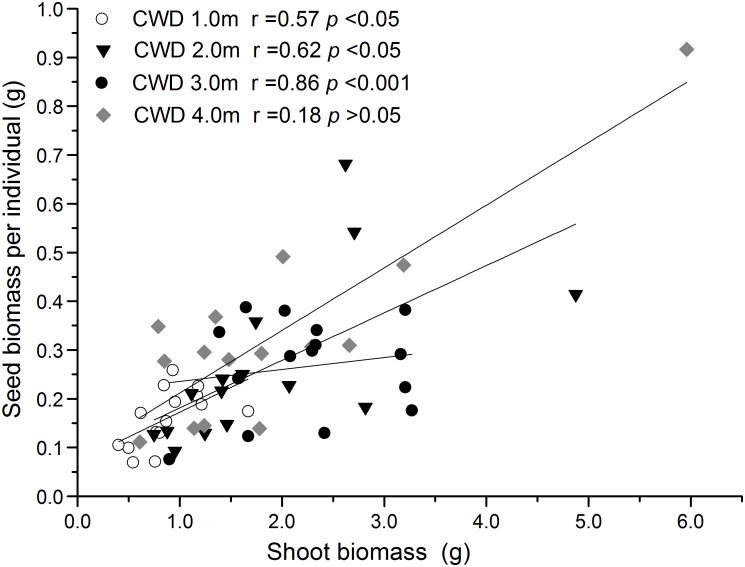
Linear correlation analysis between seed biomass and shoot biomass in different water depth environments. The significant positive correlation in 1 to 3 m depth disappeared in the 4-m depth.

The reproductive allocation characters of the individuals from different CWDs were shown in Table 1. Pollen amount per individual was positively correlated with single seed weight (Table 2), and these two characters increased significantly with water depth (Figures 3A,B). However, single seed weight was negatively correlated with seed amount, which was positively correlated with seed biomass per individual (Table 2). The inflorescence number reduced significantly along with increase of water depth (Figure 3G). This is because the individuals in deep water have fewer branches where the inflorescence is formed. Both single seed weight and seed amount determined seed biomass, which showed an inverse variation trend of shoot biomass (Figures 3F,H). There were significant variations in most sexual characters among different CWDs except seed set (Figure 3). To investigate the relationship between sexual and asexual reproduction, seeds and shoot biomass per individual were analyzed by correlation analysis and linear regression (Figure 4). We found that there were positive correlations between seeds and shoot biomass in CWD1, CDW2, and CDW3, but such correlations disappeared in CWD4 (*r* = 0.18, *p* > 0.05).

**Table 1:** Reproductive allocation characters among different water depths (means ± standard error, in each CWD *n* = 15).

Water depth	Seed set (%)	Shoot biomass (g)	Pollen amount per inflorescence	Inflorescence number	Seed biomass per individual (g)	Seed amount per individual	Single seed weight (mg)	Seeds/shoot ratio (%)
CWD1	39.72 ± 3.95	0.81 ± 0.08	63253.33 ± 1001.82	8.53 ± 0.29	0.16 ± 0.01	11.87 ± 1.07	13.64 ± 0.30	3.93 ± 0.27
CWD2	28.89 ± 3.24	1.70 ± 0.25	68949.33 ± 2762.78	5.4 ± 0.19	0.26 ± 0.04	20.93 ± 3.34	12.63 ± 0.30	3.67 ± 0.33
CWD3	27.5 ± 4.44	1.89 ± 0.33	91810.13 ± 3257.66	2.93 ± 0.18	0.36 ± 0.05	26.2 ± 4.20	13.95 ± 0.40	3.09 ± 0.30
CWD4	35.56 ± 3.61	2.06 ± 0.17	93888 ± 3314.80	2.0 ± 0.53	0.27 ± 0.03	13.93 ± 1.62	20.22 ± 0.90	2.68 ± 0.29


**Table 2:** Pearson correlation analysis among the eight characters.

	Seed set	Pollen amount per inflorescence	Seed biomass per individual	Single seed weight	Seeds/shoot radio	Inflorescence number	Seed amount per individual
Pollen amount per inflorescence	*r* = -0.154						
Seed biomass per individual	*r* = -0.055	*r* = 0.223					
Single seed weight	*r* = -0.189	*r* = 0.465^∗∗^	*r* = -0.069				
Seeds/shoot radio	*r* = 0.013	*r* = -0.141	*r* = 0.218	*r* = -0.295^∗^			
inflorescence number	*r* = 0.239	*r* = -0.708^∗∗^	*r* = -0.360^∗∗^	*r* = -0.505^∗∗^	*r* = 0.358^∗∗^		
Seed amount per individual	*r* = -0.012	*r* = 0.073	*r* = 0.964^∗∗^	*r* = -0.293^∗^	*r* = 0.260^∗^	*r* = -0.207	
Shoot biomass	*r* = -0.049	*r* = 0.178	*r* = 0.715^∗∗^	*r* = 0.112	*r* = -0.408^∗∗^	*r* = -0.471^∗∗^	*r* = 0.654^∗∗^


*^∗^*p* < 0.05; ^∗∗^*p* < 0.01; *n* = 60.*

### *In situ* Germination Experiment

#### Environmental Factors

**FIGURE 5:**
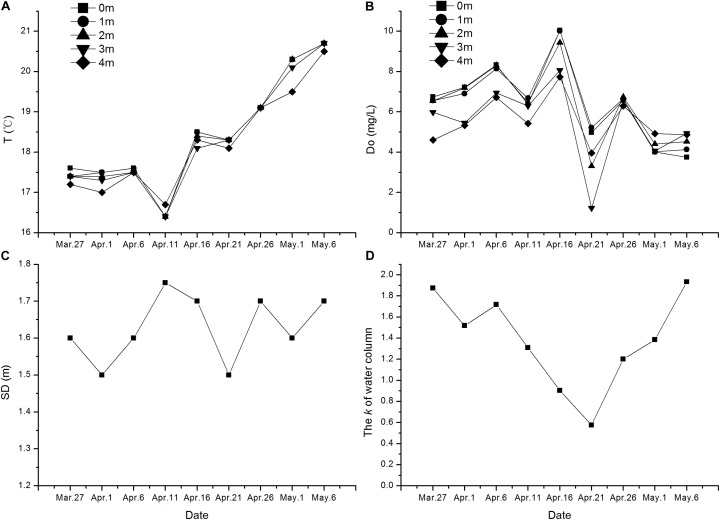
The variation of environmental characters during the germination experiment. **(A)** Temperature of water, **(B)** Dissolved oxygen, **(C)** Secchi depth, and **(D)** Extinction coefficient of water.

According to our results of the four environmental factors measured, there is no stratification in the water column of the experimental area. Environmental factors during the experimental period are shown in Figure 5. The SD varied from 1.50 m to 1.75 m during the experiment with an average of 1.63 m. Water temperature increased from 17.40°C to 21.00°C at the end. Dissolved oxygen in water column changed a little greatly, and the mean value is 5.95 mg/L. The extinction coefficient of water was related to SD. Most of the environmental factors were relatively stable during the experiment.

The current velocity of the transaction and shoreline area are shown in the Appendix. The average speed in shoreline area (water depth between 1 m and 6 m) was 0.20 m/s, and there was no significant variation in current velocity in this area.

#### Germination Results

**FIGURE 6:**
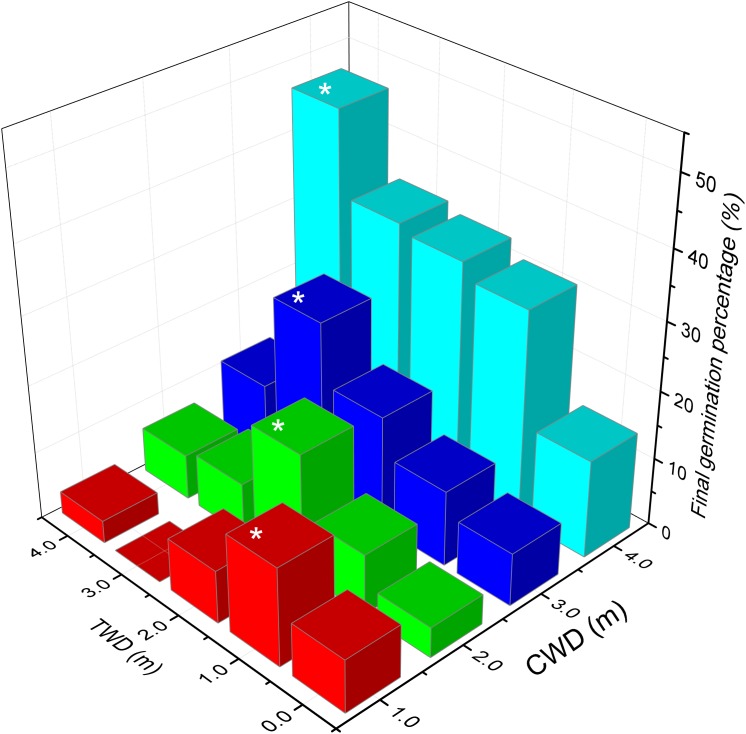
The final germination percentage of the *in situ* germination experiment. Different colors present different CWD groups. The asterisk indicates highest germination percentage of each CWD group (*p* < 0.001).

The highest final germination (46.65%) was found in the treatment CWD4×TWD4, while the lowest germination (0%) was found in CWD1× TWD3 (Table 3, Figure 6). In general, CWD4 seeds present the highest germination percentage in every TWD. Both CWD and TWD dramatically affect the final germination percentage (Table 4).

**Table 3:** Final germination percentage of the *in situ* germination experiment (means ± standard error, *n* = 3).

		CWD			
		
		1.0 m	2.0 m	3.0 m	4.0 m
TWD	0.0 m	7.78 ± 1.11 bc	4.44 ± 1.11 b	7.78 ± 1.11 c	14.44 ± 1.11 c
	1.0 m	14.4 ± 1.1 a	8.88 ± 1.92 b	13.32 ± 1.11 bc	31.09 ± 1.02 b
	2.0 m	9.99 ± 1.92 b	17.75 ± 1.11 a	16.64 ± 1.92 b	33.32 ± 1.93 b
	3.0 m	0 ± 0 d	7.77 ± 1.92 b	25.51 ± 1.09 a	34.44 ± 1.1 b
	4.0 m	4.44 ± 1.1 bd	6.66 ± 1.09 b	11.11 ± 1.11 bc	46.65 ± 1.92 a


*Different letters indicate significant variation in each CWD group (*p* < 0.05).*

**Table 4:** Two-way ANOVA results of the final germination percentage.

Source of variation	*df*	SS	MS	*F*	*P*
TWD	4	766.596	191.649	35.683	<0.001
CWD	3	5882.704	1960.901	365.096	<0.001
TWD × CWD	12	2083.865	173.655	32.332	<0.001
Error	40	214.837	5.371		
Total	60	23466.822			


The maximum germination percentages of seeds from CWD1, CDW2, CDW3, and CDW4 are 14.4%, 17.75%, 25.51%, and 46.65%, respectively. In the same CWD, there is a significant differentiation in final germination percentage according to different TWDs. Seeds exhibited maximum germination percentage only when TWD was consistent with CWD (Figure 6). For instance, CWD4 seeds have the highest germination percentage (46.65%) only in TWD4 condition. Similarly, CWD3 seeds exhibit the highest germination percentage (25.51%) only in TWD3 condition. In other words, the most suitable germination depth is the same as its growing depth.

In each TWD, seeds from the deepest water habitat (CWD4) always represent highest germination percentage (14.44%, 31.09%, 33.32%, 34.44%, and 46.65% for TWD-0, TWD1, TWD2, TWD3, and TWD4, respectively). Another interesting result was that in every TWD, the seeds from deeper water showed higher final germination percentage than those from shallower water. When we separated the data by different CWDs, the average final germination percentage increased with CWD. This means that the seeds coming from deeper habitats have higher germination ability, which could be treated as seed quality. When we considered the variation of seed amount (quantity) in different water depths, such a tendency could be treated as a trade-off between seed quantity and quality depending on water depth.

### Half Final Germination Time and Potential Germination Depth

**FIGURE 7:**
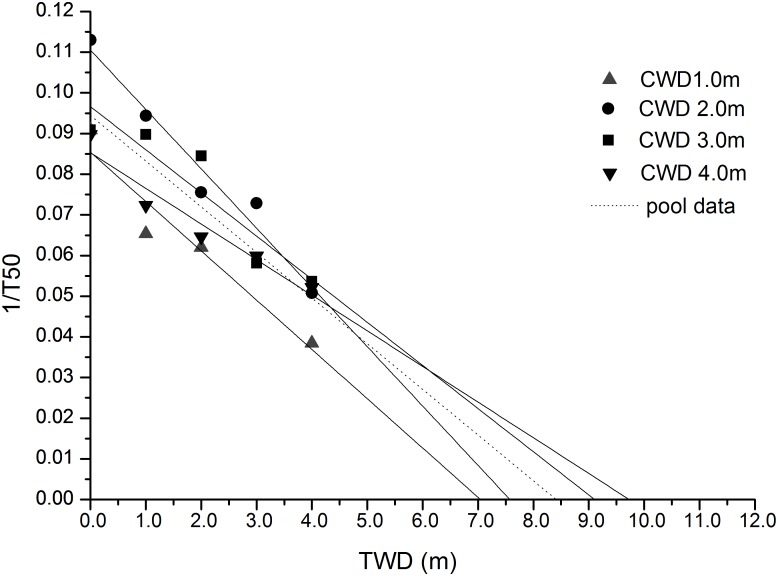
Linear regression analysis between TWD and 1/*T*_50_.

Most of the seeds completed germination within 20 days from sowing, but germination speed varied among the CWDs. The average *T*_50_ of the four CWD seeds was 14.77 days (Table 5). The highest (26.00 days) was found in the CWD1 seeds germinated in TWD4, while the lowest was 8.85 days, found in CWD2 seeds germinated in TWD0. Based on 1/*T*_50_ and TWD, a linear analysis was performed (Figure 7). From the intersection with the TWD axis, we got the PMGD for seeds from each CWD and also for total seeds.

**Table 5:** Half final germination time (*T*_50_) of the *in situ* germination experiment (days).

	CWD1	CWD2	CWD3	CWD4
TWD0	11.00	8.85	11.00	11.14
TWD1	15.29	10.59	11.14	13.82
TWD2	16.13	13.23	11.83	15.47
TWD3		13.72	17.20	16.70
TWD4	26.00	19.69	18.64	19.16


## Discussion

### Trade-Offs in Different Aspects

Both sexual and asexual reproduction are important to aquatic plants, and the resource allocation strategy between sexual and asexual reproduction has been discussed for a long time (Philbrick and Les, 1996) and evaluated in many aquatic plants (Geber et al., 1992; Prati and Schmid, 2000; van Kleunen et al., 2002; Thompson and Eckert, 2004; Liu et al., 2009; van Drunen and Dorken, 2012; Eckert et al., 2016). However, almost no related findings were reported in submerged aquatic plants until now. Our results indicated trade-offs not only between sexual and asexual reproduction but also between different sexual characters, and the existence of trade-offs is related to habitat difference.

Trade-offs are based on the theory that there is a limitation in the resources allocated to different strategies. Although trade-offs between sexual and asexual reproduction in plants are widely expected, they are less consistently found. One of the reasons is that trade-offs are usually obscured by variation in resource acquisition among individuals, which usually cause positive covariation (Eckert et al., 2016). At the species scale, we found a strong positive covariation between seeds and shoot biomass (Table 2). However, when we consider different CWDs, the relationship changed. The positive correlation was presented in CWD1 to CDW3, but there was no correlation in CWD4 (Figure 4). This implies that resource acquisition could be limited in this stressful environment and induces the breakdown of the positive correlation. A transition from positive to negative covariation is to be expected in deeper, more stressful water.

According to our results, water depth will significantly affect almost all the characters of this submerged species except seed set (Figure 3) and shape the whole population into different groups (Figure 4). We found that seeds/shoot ratio decreased dramatically with water depth (Figure 3D). This result means that allocation in sexual reproduction is going to be reduced when water depth stress increases. Based on our observations, the old shoots sink to the bottom of the lake at the end of the growing season, and new roots and shoots will sprout from each node of the old shoots at the beginning of the next growing season. So, the biomass of the shoot (without sexual organs) can be used as an indicator of allocation to asexual reproduction. Our results are consistent with the former conclusion that along the water depth gradient, sexual reproduction proportion will be reduced (Hutchinson, 1975; Philbrick and Les, 1996; Li, 2014).

Furthermore, when we consider detailed aspects of sexual reproduction, we found that both single inflorescence pollen amount and single seed weight increased dramatically from CWD1 to CWD4, indicating that resources allocated to a single sexual unit is increased. This seems to be in conflict with our former conclusion. But considering the decrease of sexual unit numbers (inflorescence number and seed amount per individual) in deeper CWD (Figure 3G), the total resource allocation in sexual reproduction is still reduced. These results illustrated a trade-off between quality and quantity of sexual reproductive units. This species tends to produce fewer but higher quality sexual units when it is facing more stressful water depth, while it tends to form more but lower quality sexual units in relatively less stressful conditions.

### Adaptive Transgenerational Plasticity in *P. maackianus*

Germination was significantly affected by water depth. But our results cannot support the second hypothesis that the lowest germination percentage is expected to be found in the deepest water habitat. However, we found a novel pattern in seed germination under water depth stress of this species (Figure 6). The maximum final germination percentage occurred only when the TWD was the same as the CWD. In other words, the water depth where seeds matured was the most suitable germination condition. Such results have never been reported before in aquatic plants. The most reliable reason for this phenomenon is adaptive transgenerational plasticity. The adaptive transgenerational plasticity was initially considered as a noise of evolution, while three decades ago it was reconceptualized as a potential source of ecologically and evolutionarily meaningful variations (Roach and Wulff, 1987; Schmitt et al., 1992; Sultan, 1996; Donohue and Schmitt, 1998). The central meaning of adaptive transgenerational plasticity was summarized as follows: parent individuals alter specific developmental traits in progeny in response to particular environmental stresses to enhance offspring fitness under the same stresses, and it can be expected to evolve if parental habitats reliably predict their offspring habitats (Agrawal et al., 2001; Galloway, 2005; Herman and Sultan, 2011).

Water depth stress is an important environmental challenge that aquatic plants (especially submerged plants) must confront. In deeper habitats, submerged plants need to change resource allocation strategy, which will not only represent in plant phenotypes but also population genetic structures and will lead to community succession (Barko and Smart, 1981; Strand and Weisner, 2001; Li et al., 2004; Yang et al., 2004; Fu et al., 2012). If the water depth of the offspring’s habitat could be predicted, it is crucial for parent individuals to maximize the fitness of their offspring in the likely habitat. As we observed, most seeds of *P. maackianus* will sink to the bottom with their maternal individuals. Considering the relative slow current velocity in the vegetation area of the lake (Appendix), we can expect that the dispersal distance of seeds of this species is very limited. Other research on population spatial genetic analysis of a related species, *P. pectinatus*, revealed that the seedling recruitment distance is less than 5 m (Triest and Fenart, 2014). Based on the underwater topographic map of Erhai Lake, the lake bed depth varies slowly on the west side, but the east side is cliffy. This species is distributed mainly in the western part of the lake. The distances between every two adjacent CWDs in the bays dominated by this species are 10.80 m to 298.00 m, 13.60 m to 265.00 m, and 24.00 m to 589.30 m for CWD12, CWD23, and CWD34, respectively. This means that the dispersal distance of seeds is smaller than the scale of environmental heterogeneity within a population of this species. In this condition, adaptive plasticity between generations could be evolved to enhance offspring fitness in the predictable similar environment (Galloway, 2005). Our results clearly illustrate this opinion.

This research is the first attempt to directly prove the adaptive transgenerational plasticity in a typical submerged macrophyte. We can speculate that the original population of *P. maackianus* will gradually separate into different groups according to water depth. Considering our results, in each group, seed fitness is highest in the parental habitat. This will accelerate the fixation of specific adaptive characters and result in a lot of change in population genetic structure, which will play an important role in the evolutionary dynamics of populations (Rasanen and Kruuk, 2007).

The mechanism for these remarkably specific effects of parental environment on seedling growth patterns may be found in the fact that content and balance of growth hormones in seeds is affected by many aspects of the parental environment, including drought, mineral nutrient supply, light quality and duration, and temperature (Gray and Thomas, 1982; Gutterman, 1982). But more evidence from different aspects (physiological, ecological, genetic, etc.) is needed to reveal the mechanisms of this phenomenon and its evolutionary meaning at ecological timescales.

### Potential Maximum Germination Depth

According to our results, the seeds from CWD2 and CWD3 represent higher germination speed but were affected by germination water depth significantly. While seeds from CWD4 failed to show a high germination speed, they represented some resilience to water depth. Based on the linear regression between TWD and 1/*T*_50_, we can determine the threshold water depth over which germination of seeds almost does not take place, and this water depth is defined as PMGD. In practice, this model can be used to predict the germination dynamics of seeds simply from the mean water depth of a given lake, as long as the light attenuation characteristics are constant. In our research, we can get the PMGD for *P. maackianus* seeds in this lake, when we use all the data for calculation.

The aquatic vegetation in many freshwater lakes, including Erhai Lake, being affected by eutrophication, was reduced dramatically. From the 1990s, the aquatic vegetation coverage in this lake dropped from 40% to 10% (Fu et al., 2013). Considering the important ecosystem functions of submerged macrophytes, submerged vegetation restoration is arising as an important task for many eutrophicated lakes. Since the 1990s, *P. maackianus* has become the dominant species in this lake, and there was once a large population of this species in the south central part of the lake before 1998 (Fu et al., 2013). According to historical records, the maximum water depth in this large population was about 7–8 m. This is very close to our PMGD result. Based on our results, a supply of ripe seeds of *P. maackianus* into this area could be helpful in vegetative restoration. Ripe seeds collected from 4-m deep or deeper habitats are more recommended. Our research provided a solid support to the usage of seeds for aquatic vegetation restoration.

## Author Contributions

XZ and HS conceived the study. TZ and XB assisted the experiments. HS and XZ analyzed the data. HS, LN, PX, and XZ wrote and revised the paper.

## Conflict of Interest Statement

The authors declare that the research was conducted in the absence of any commercial or financial relationships that could be construed as a potential conflict of interest.

## Figures and Tables

**APPENDIX A1:**
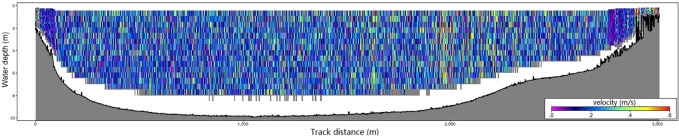
The current velocity of a transection in Haichaohe bay.

**APPENDIX A2:**
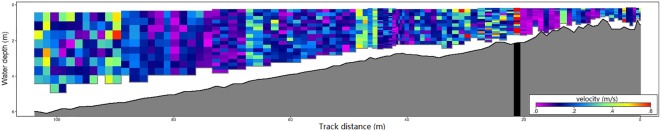
The current velocity in the west bank of the transection.

**APPENDIX A3:**
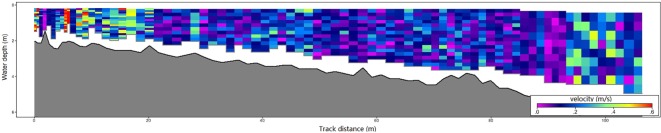
The current velocity in the east bank of the transection.

## Abbreviations

CWD: collection water depth
PMGD: potential maximum germination depth
TWD: treatment water depth
